# Antimicrobial Properties and Cytotoxicity of LL-37-Derived Synthetic Peptides to Treat Orthopedic Infections

**DOI:** 10.3390/antibiotics13080764

**Published:** 2024-08-14

**Authors:** Vincenzo Pennone, Elisa Angelini, David Sarlah, Arianna B. Lovati

**Affiliations:** 1 Cell and Tissue Engineering Laboratory, IRCCS Istituto Ortopedico Galeazzi, 20157 Milan, Italy; arianna.lovati@grupposandonato.it; 2 Department of Chemistry, University of Pavia, 27100 Pavia, Italy; elisa.angelini01@universitadipavia.it (E.A.); sarlah@unipv.it (D.S.); 3 Department of Chemistry, Carl R. Woese Institute for Genomic Biology and Cancer Center at Illinois, University of Illinois, Urbana, IL 61801, USA

**Keywords:** synthetic peptides, LL-37, antimicrobial activity, biocompatibility, cytotoxicity, orthopedic infections

## Abstract

Open fractures and prosthetic joints are prone to bacterial infections, especially those involving biofilms, and are worsened by antibiotic inefficacy and resistance. This highlights the need for targeted treatments against orthopedic infections. LL-37, a human cathelicidin, is known for its antimicrobial properties. This study aimed to synthesize and evaluate LL-37-derived antimicrobial peptides (AMPs) for antibacterial efficacy and toxicity. Several truncated LL-37 analogues were created and tested against 18 bacterial strains, both ATCC and orthopedic clinical isolates, using MIC and MBC assays. Synergy with antibiotics and resistance development were also analyzed, alongside cytotoxicity on NIH-3T3 fibroblasts and hemolytic activity assessments. Six AMPs were synthesized, with FK-16 and GF-17 emerging as the most effective. The MIC values ranged from 4.69 to 18.75 µg/mL and 2.34 to 18.75 µg/mL, respectively, against *S. epidermidis* and *S. aureus*, with the MBC values matching the MIC values. Cytotoxicity tests showed no toxicity at concentrations below 75 µg/mL for GF-17 and 150 µg/mL for FK-16. Hemolytic activity was below 1% at 18.75 µg/mL for GF-17 and 75 µg/mL for FK-16. These AMPs showed no synergistic effects with antibiotics and no resistance development. FK-16 and GF-17 effectively removed biofilms, particularly against *S. epidermidis*. Incorporating these AMPs into surgical materials (hydrogels, cements, etc.) could enhance infection control in orthopedic procedures, warranting further in vivo studies.

## 1. Introduction

The prevalence of bacterial infections in orthopedics, mainly implant-related infections, open fractures and prosthetic joints, represents a critical clinical challenge due to a high susceptibility to bacterial infections, and particularly due to the propensity of these infections to form biofilms. Biofilms significantly contribute to the resilience of bacterial communities against conventional antibiotic treatments, leading to enhanced antibiotic resistance [1] and complicating the management of orthopedic infections [2]. This resistance is largely attributed to the protective environment that biofilms provide to bacteria, shielding them from both immune responses and antibiotic penetration, and is exacerbated by the growing issue of antibiotic resistance. These regions also receive a limited blood supply, particularly in the presence of comorbidities (cardiovascular disorders, diabetes, etc.) [3,4], which restricts the reach of systemically administered antibiotics. This situation is exacerbated by the unique interactions of bacteria with implant surfaces, often resulting in chronic infections that are difficult to eradicate and may necessitate surgical intervention [5]. The failure of standard antibiotics to effectively manage these biofilm-associated infections highlights an urgent need for innovative therapeutic strategies, in particular in the presence of antimicrobial-resistant strains. 

Antimicrobial peptides (AMPs) have emerged as promising candidates due to their potent efficacy against biofilm-forming bacteria commonly encountered in orthopedic settings, including *S. aureus* and *S. epidermidis* [2], and AMPs exhibit a low potential to promote drug resistance [6]. AMPs can be either synthetically produced or derived through recombinant techniques [7], providing versatile platforms for designing targeted anti-infective therapies. Among AMPs, LL-37—a naturally occurring human cathelicidin secreted in a variety of cells and body fluids in the human body—is one of the most studied in the literature [8]. Unlike traditional antibiotics, LL-37 and its derivatives have been shown to disrupt biofilms, offering potential not only for treating existing infections but also for preventing bacterial colonization on prosthetic materials [5]. Given its broad-spectrum activity and mechanism that differs significantly from traditional antibiotics, LL-37 and its derivatives present a promising alternative in the fight against resistant infections associated with orthopedic procedures. However, it has been noted that LL-37 induces hemolysis and exhibits toxicity towards human leukocytes and the T-lymphocytes, likely because of hydrophobic interactions with eukaryotic cell membranes [9,10,11]. Moreover, human LL-37, comprising 37 amino acids, can be expensive to produce chemically due to its length. Thus, LL-37 fragments can be successfully synthesized to identify and select analogs with higher antimicrobial and antibiofilm potential [12,13].

Based on extensive structure–activity relationship studies involving LL-37 [12,14,15,16], we chose several shorter fragments that are known to mimic the structural features of the parent lead LL-37. The selection of truncated analogs was conducted by way of an analysis of their biological activities, with a focus on a diverse range of peptides that are known to have antibacterial activities as well as activity to prevent biofilm development [17]. The development of LL-37 derivatives represents a significant advancement in antimicrobial peptide research. These peptides offer a balanced combination of potency, stability and reduced cytotoxicity, making them promising candidates for future therapeutic applications [5,18,19,20,21]. However, the effectiveness of these peptides can vary depending on the specific pathogen and its resistance mechanisms. This variability necessitates a tailored approach for different clinical scenarios, such as their use in orthopedics. Accordingly, we prepared KR-12, FK-13, FK-16, GF-17, 17BIPHE2 and SK-24 as a representative library of native and modified shorter sequences of LL-37. For example, KR-12 corresponds to residues 18–29 of LL-37 and FK-13 (residues 17 to 29) displayed antibacterial activity with minimal toxicity towards human cells [22]. Similarly, the peptide FK-16 (residues 17 to 32) and its N-glycinated variant, GF-17, have been shown to exhibit strong bactericidal activity against a host of ESKAPE organisms [5,13]. On the other hand, 17BIPHE2 is an engineered analog of GF-17, where two L-isoleucines and one L-leucine are replaced with corresponding D-leucines and two phenylalanines are also replaced by two biphenylanines to improve stability towards protease degradation [23]. Finally, SK-24 corresponds to the long hydrophobic domain (residues 9–32) and has shown an antimicrobial spectrum comparable to that of LL-37 [21].

This study aimed to synthesize and evaluate in vitro the efficacy and safety of several LL-37-derived synthetic AMPs. Utilizing Fmoc solid-phase peptide synthesis, a series of truncated analogues of LL-37 was generated. The antimicrobial activity of these peptides was tested against a panel of bacterial strains relevant to orthopedic infections, including both ATCC reference strains and clinically isolated strains. Furthermore, this study investigated the potential synergistic effects of these AMPs when combined with conventional antibiotics and assessed the in vitro antimicrobial resistance potential and cytotoxicity to ensure the safety of the peptides for potential in vivo studies before clinical use. This research underscores the potential of integrating these AMPs into materials used in surgical settings to enhance infection control measures in orthopedic procedures, but also to identify a potential pathway to address existing antibiotic resistance.

## 2. Results

### 2.1. Synthesis and Purification of AMPs

The LL-37-derived antimicrobial peptides to be synthesized and tested were first chosen based on the literature reports about their structure–activity relationships [24] before evaluating the possibility of synthesizing novel compounds. The chosen peptides, shown in Table 1, were KR-12 [25], FK-13 [19], FK-16 [26], GF-17 [27], 17BIPHE2 [16] and SK-24 [21], respectively. All the selected sequences are known, biologically active, shorter fragments of the native peptide, except 17BIPHE2, a modified version of GF-17 containing D-Leucine and biphenylalanine replacements. All of them were synthesized as TFA salts with ≥95% purity using standard Fmoc solid-phase peptide synthesis (SPPS) and HPLC chromatography for purification.

### 2.2. Screening of Synthetic AMPs for Antimicrobial Activity against ATCC Strains

A first screening was performed by assessing the MIC and MBC against ATCC strains of all six AMPs, as well as a commercial synthetic LL-37 (cLL-37) and Na-TFA. The results reported in Table 2 showed that cLL-37 exhibited MIC values ranging from 9.38 µg/mL to 75 µg/mL. However, these concentrations did not achieve bactericidal effects for all isolates, as indicated by the MBC values (Table 2). In particular, *S. epidermidis* ATCC14990, *P. aeruginosa* ATCC27853 and *E. coli* ATCC25933 were inactivated at a concentration of 75 µg/mL, while the remaining ATCC strains were still viable at a concentration of 300 µg/mL. FK-16 and GF-17 AMPs achieved the best performance due to their bacteriostatic and bactericidal activities against almost all the strains tested (Table 2). In particular, against the genus *Staphylococcus*, FK-16 showed inhibition at concentrations ranging from 4.69 to 18.75 µg/mL, while GF-17 showed inhibition at 2.34 to 4.69 µg/mL. The *E. coli* strain was inhibited at concentrations of 9.38 and 18.75 µg/mL by GF-17 and FK-16, respectively, while FK-16 had a MIC of 150 µg/mL and GF-17 18.75 µg/mL (Table 2) against *P. aeruginosa*. The bactericidal effect of FK-16 against *Staphylococci* ranged between 9.38 and 75 µg/mL, while it was 75 µg/mL for *E. coli*. Against *P. aeruginosa*, FK-16 had a bactericidal effect only at 300 µg/mL. GF-17 had a bactericidal effect against *Staphylococci* at concentrations ranging from 4.69 to 150 µg/mL. Against *E. coli* ATCC 25922, the MBC of GF-17 was 300 µg/mL—much higher than the MIC value of 9.38 µg/mL. Eventually, GF-17 had a bactericidal effect against *P. aeruginosa* at 75 µg/mL. 17BIPHE2, the D-LEU analog of GF-17, and SK-24 showed a similar bacteriostatic effect on *Staphylococci* strains, while 17BIPHE2 had a lower MIC against *P. aeruginosa* ATCC 27,853 and a lower MIC and MBC against *E. coli* ATCC 25,922 compared to SK-24. KR-12 did not inhibit the growth of any tested strain as its MIC values were consistently greater than 300 μg/mL for all strains. FK-13 exhibited antimicrobial activity at high concentrations, with MIC values ranging from 75 μg/mL to over 300 μg/mL. However, FK-13 did not achieve bactericidal effects as the MBC values were consistently higher than 300 μg/mL for all strains. For control, vancomycin was tested against *S. aureus* 43,300 and showed a MIC and MBC of 0.6 μg/mL, categorizing the strain as susceptible (S, according to EUCAST clinical breakpoints tables, version 14), while it was confirmed as ineffective against gram negative strains. Gentamicin showed MICs of 0.6 μg/mL against *S. aureus* 43,300 (S), *P. aeruginosa* 27,853 (S) and *E. coli* 25,922 (S), while its MBCs were 2.5 μg/mL. Finally, no bacteriostatic or bactericidal effect was shown by Na-TFA at the concentrations tested.

### 2.3. Biocompatibility Evaluation on Eukaryotic Cells of Selected Synthetic AMPs

**Figure 1:**
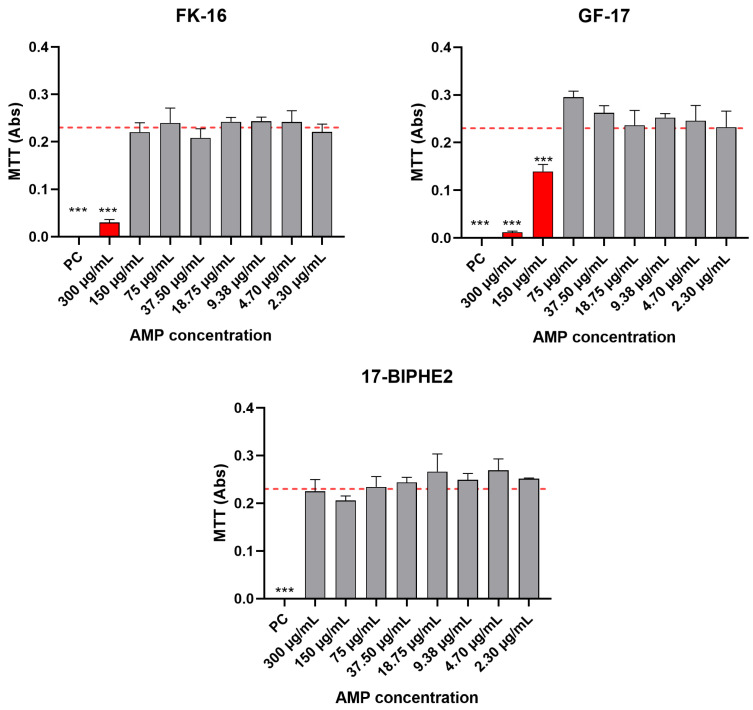
Effect of selected AMPs at different concentrations on the viability of NIH-3T3 fibroblast compared to cells cultured in CM (NC) and CM supplemented with 0.1% Triton-X100 (PC) assessed by means of the MTT assay. Red dotted lines represent the NC mean value of Abs (0.23). Statistical significance: *p* < 0.0001 (***).

After verifying the antibacterial efficacy of the synthesized AMPs, only FK-16, GF-17 and its analogue, 17BIPHE2, demonstrated significant antimicrobial properties. Therefore, these three peptides were included in the cytotoxicity studies to assess their safety in vitro, which is essential for future in vivo studies. This selection was based on their highest antimicrobial activity, ensuring that only the most effective and promising candidates were further evaluated for biocompatibility. The biocompatibility of the selected AMPs was assessed using the spectrophotometric MTT viability assay. No signs of cytotoxicity were recorded when culturing NIH-3T3 fibroblasts in the presence of FK-16, GF-17 and 17BIPHE2 for AMP concentrations of 150, 75 and 300 µg/mL and lower, respectively, as illustrated in Figure 1. Cells cultured with AMPs at the aforementioned concentrations had comparable absorbance levels to cells cultured in fresh complete medium (CM = NC). Therefore, the statistical differences reported in the histogram refer to the comparison between NC and AMP concentrations that are equal to the PC (positive control). The mitochondrial function assessment by the MTT assay can work as an index to estimate the relative cell number to NC, and it reached 100% of cell viability when cells were cultured in the presence of specific AMP concentrations. 

### 2.4. Hemolytic Activity of Selected Synthetic AMPs

Drugs are often required to meet stringent criteria for hemolysis, which may include limits of less than 1% or even lower. The limits are established to guarantee patient safety and the effectiveness of the drug [28]. 

**Figure 2:**
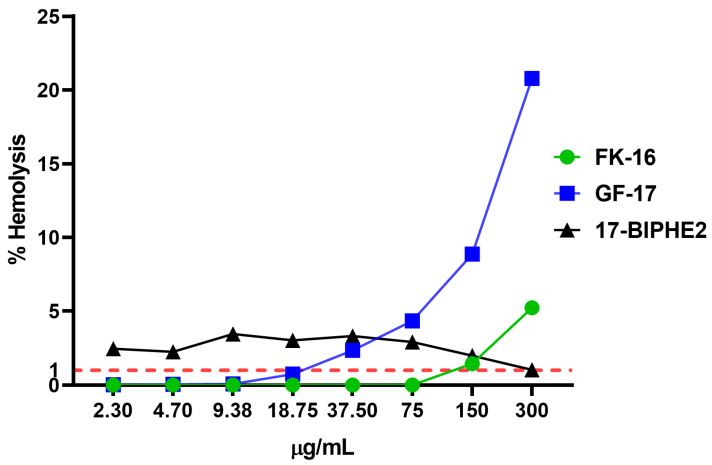
Hemolytic activity expressed as a percentage (%). Red dotted line reports the cut off range as ≤1%.

In Figure 2, the results of hemolytic activity experiments show that FK-16 had hemolytic activity below 1% at a concentration of 75 µg/mL and GF-17 at a concentration of 18.75 µg/mL, while 17BIPHE2 was hemolytic at any concentration. For this reason, 17BIPHE2 was not included in further investigations. No hemolytic activity was observed in TFA.

### 2.5. MIC and MBC of Selected AMPs against Clinical Isolates

Similar to the results obtained with ATCC strains, GF-17 and FK-16 showed higher antimicrobial activity against clinical isolates, with lower MICs and MBCs compared to cLL-37 (Table 3). In particular, against *Staphylococci,* GF-17 and FK-16 showed lower MIC values than cLL-37, with GF-17 being lower than FK-16. These AMPs also showed higher bactericidal activity than cLL-37 against *S. aureus* strains, with lower MBCs. On the contrary, against *P. aeruginosa* clinical isolates, neither of the two AMPs performed better than cLL-37. Eventually, against *E. coli* clinical strains, FK-16 and GF-17 had the same antimicrobial effect and FK-16 had a slightly better bactericidal effect compared to GF-17, but in both cases, the two AMPs performed better than cLL-37 (Table 3). As a control, vancomycin and gentamicin were tested against *S. epidermidis* GOI1153754-03-14, which proved susceptible against vancomycin and resistant to gentamycin (S and R, respectively, according to EUCAST clinical breakpoints tables, v. 14).

**Figure 3:**
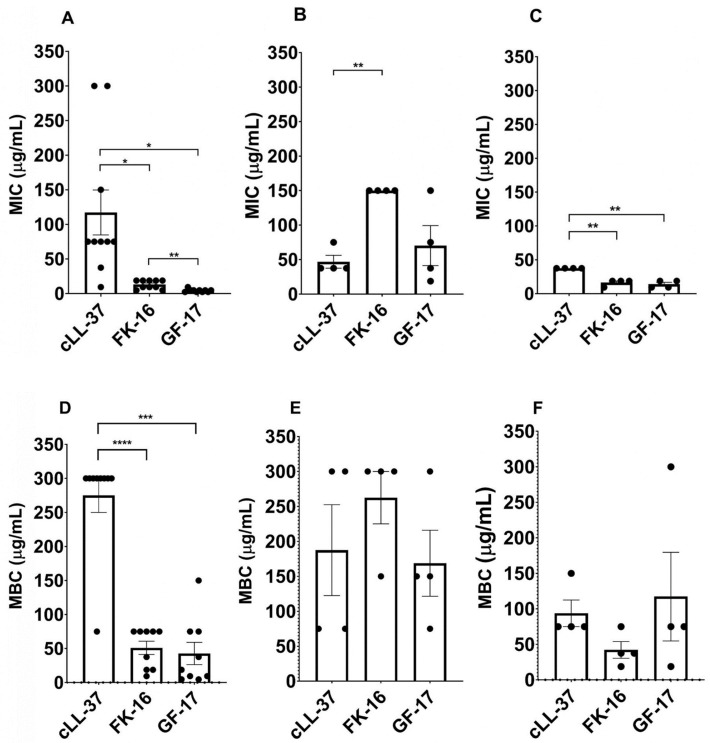
MIC (**A**–**C**) and MBC (**D**–**F**) values comparing cLL-37, GF-17 and FK-16. Data were grouped per bacterial genera: Staphylococci (**A**,**D**), *P. aeruginosa* (**B**,**E**), *E. coli* (**C**,**F**) and reported as mean ± SEM. Significance, where present, is shown. * *p* ≤ 0.05; ** *p* ≤ 0.01; *** *p* ≤ 0.001; **** *p* ≤ 0.0001.

Merging the results from ATCC and clinical isolates, MIC and MBC data showed significant differences that are summarized in Figure 3. In detail, in the case of *Staphylococci*, both FK-16 and GF-17 produced MIC and MBC values significantly lower than cLL-37, and GF-17 had MIC values significantly lower than FK-16 (*p* < 0.05, Figure 3A,D). Against *P. aeruginosa*, FK-16 showed MIC values significantly higher than cLL-37 (*p* < 0.05, Figure 3B) and no difference in MBC values (Figure 3E), while, against *E. coli* strains, FK-16 and GF-17 had MIC values significantly lower than cLL-37 (*p* < 0.05, Figure 3C,F). 

### 2.6. Synergy between Selected AMPs and Vancomycin

This experiment was conducted to assess whether there was a synergy between vancomycin and the two best-performing AMPs, FK-16 and GF-17, followed by a combination of the two selected AMPs, FK-16 and GF-17. As shown in Table 4, none of the experiments showed a FIC value below 0.5, indicating no synergistic effects. Despite this, the combination of GF-17 and FK-16 exhibited additive effects, with FIC values ranging from 0.5 to 1.

### 2.7. Selection for Resistance

**Figure 4:**
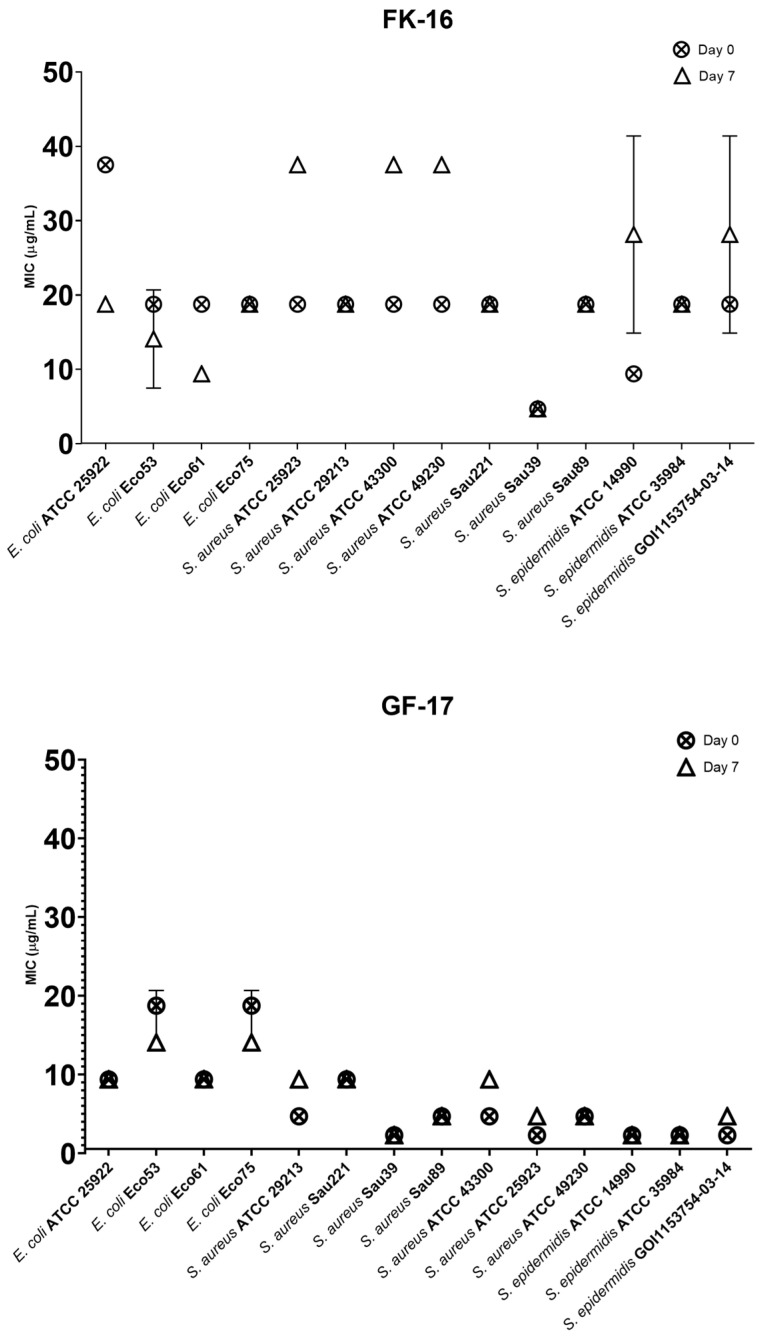
Changes in the MIC values of FK-16 and GF-17 on different strains.

The *P. aeruginosa* strains were excluded from this study because the MIC values of FK-16 and GF-17 against these strains exceeded the concentrations at which hemolysis occurred in the test described in Table 4. Only some ATCC strains and the clinical isolate MRSE-GOI1153754-03-14, tested against FK-16, increased their MIC values at Day 7, while all the other clinical isolates did not develop any resistance (Figure 4, FK-16). However, only in *S. epidermidis* ATCC14990 was this increment about fourfold higher than the MIC at Day 0. Moreover, after a further seven-day incubation in absence of FK-16, the MIC values for *S. epidermidis* ATCC14990 decreased back to the initial values. With GF-17, the clinical isolates did not modify their MIC values, while some ATCC strains increased their MIC values by only twofold (Figure 4, GF-17).

### 2.8. Antibiofilm Activity

**Figure 5:**
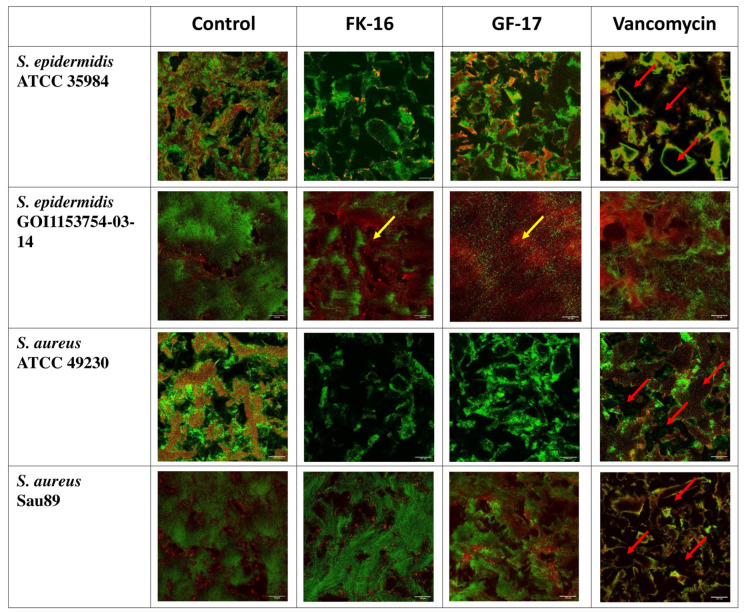
CLSM images of 72-h-old biofilms produced by *S. epidermidis* ATCC 35984 and GOI1153754-03-14, *S. aureus* ATCC 49230 and Sau89. In the columns, the untreated biofilm and biofilms after treatments with FK-16, GF-17 and vancomycin are depicted. Scale bars: 100 μm. Red arrows highlight the biofilm disruption by vancomycin, while yellow arrows show the abundance of dead (red) bacteria in the case of FK-16 and GF-17 on *S. epidermidis* GOI1153754-03-04 biofilms.

On 72-h-old biofilms, the application of the antimicrobials (FK-16, GF-17 and vancomycin) showed stain-dependent results (Figure 5). Confocal laser scanning microscopy (CLSM) observations indicated that both AMPs exhibited biofilm removal activity, especially against *S. epidermidis* strains (Figure 5). In particular, against *S. epidermidis* GOI1153754-03-14, FK-16 and GF-17 resulted in more red-stained cells (dead bacteria) than green cells (live bacteria), demonstrating their effective killing power on pre-formed biofilm. Vancomycin caused structural disruption in almost all biofilms tested but had a less disruptive effect on *S. epidermidis* GOI1153754-03-14 (Figure 5). 

**Figure 6:**
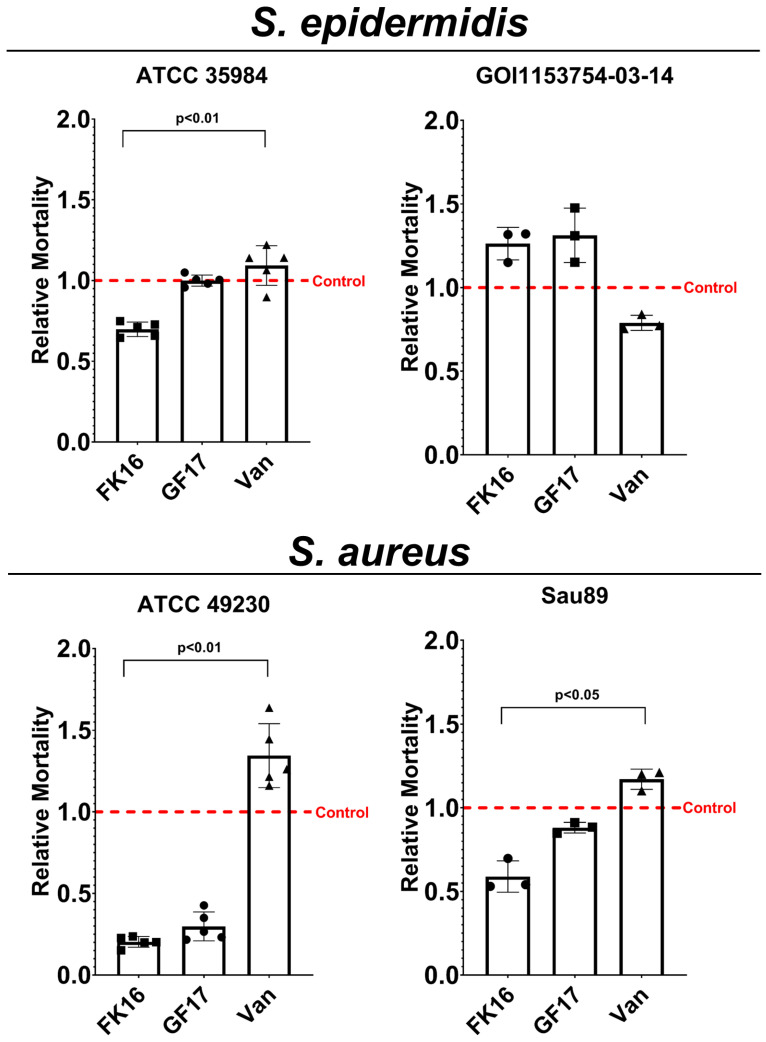
Histograms show the mean ± SD of relative mortality of each treatment in comparison with the controls (untreated). *p* values are shown where the differences were statistically significant.

Quantitative analysis of the fluorescent signals in both channels of each image confirmed the observed efficacy against *S. epidermidis* strains (Figure 6). In *S. epidermidis* ATCC 35984 biofilms, the amount of dead bacteria was higher than the uninoculated control when treated with vancomycin, and this was statistically different from the FK-16 treatment (*p* < 0.01). For *S. epidermidis* GOI1153754-03-14, FK-16 and GF-17 treatments showed more dead bacteria than vancomycin, even though the differences were not statistically significant (*p* > 0.05). In biofilms formed by *S. aureus* strains, vancomycin treatment produced more dead cells compared to FK-16 treatment (Sau89 *p* < 0.05 and ATCC 49230 *p* < 0.01, Figure 6).

## 3. Discussion

The present manuscript explores the utility of synthetic analogues of LL-37, a well-characterized antimicrobial peptide, focusing on their potential use against orthopedic infections that are complicated by the presence of biofilm-forming bacteria and antibiotic resistance. The application of AMPs in orthopedic settings represents a significant advancement in preventing and treating infections associated with orthopedic implants and surgeries. The innovative aspect of this study lies in its focus on testing LL-37-derived peptides specifically on bacterial strains isolated from orthopedic infections.

This study highlights the notable antimicrobial ability of two specific peptides, FK-16 and GF-17, which exhibit substantial inhibitory and bactericidal activity at relatively low MICs and MBCs. GF-17 differs from FK-16 only in one N-terminal glycine, which induces a higher antimicrobial potency, as demonstrated by lower MIC and MBC values in the present study. This single amino acid addition affects the peptide flexibility, charge distribution and membrane interaction dynamics [23], making GF-17 more effective in disrupting bacterial membranes compared to FK-16. The hydrophobic characteristics of GF-17 might be the explanation for its more powerful antimicrobial activity against *Staphylococci*, as shown in our results. This is particularly relevant for pathogens like methicillin-resistant *Staphylococcus aureus* (MRSA) and other multidrug-resistant organisms that pose significant challenges in clinical settings due to their robust resistance mechanisms and biofilm-forming capabilities. These findings are congruent with the existing research [2,5], which documents the efficacy of AMPs in not only inhibiting microbial growth but also disrupting established biofilms. In orthopedics, biofilm formation on implants is a critical challenge, leading to persistent infections that are difficult to treat with conventional antibiotics. Moreover, biofilm enhances bacterial resistance to antibiotics by physically blocking antibiotic penetration and facilitating a communal survival strategy among bacteria. The efficacy of FK-16 and GF-17 against such biofilm-associated infections aligns with the findings of Wei et al. [2], which demonstrated that certain AMPs could penetrate and disrupt biofilm structures, thereby enhancing the susceptibility of bacteria to antimicrobial agents. By demonstrating efficacy against such biofilm-associated infections, FK-16 and GF-17 strengthen the therapeutic potential of AMPs as effective agents in the ongoing fight against resistant infections in orthopedics. By effectively lowering the MIC and MBC values, these peptides suggest a potential reduction in the dosage requirements of conventional antibiotics when used in combination therapies, although this study did not demonstrate synergistic effects with other antibiotics. This aspect could be vital in reducing side effects and minimizing the risk of further resistance development. This finding is supported by Mishra et al. [5], who reported that combining AMPs with traditional antibiotics could lower the necessary dosages of both, potentially minimizing side effects and reducing the risk of further resistance development. However, our study did not demonstrate synergistic effects with other antibiotics, which contrasts with some previous studies [2] that found synergistic interactions between certain AMPs and antibiotics. On the other hand, Han et al. [29] showed that FK-16 and FK-13 had some synergistic effects with Penicillin G and Ampicillin, but not with other antibiotics tested. More work is needed to explore possible synergies with other antibiotics.

Moreover, the broad-spectrum activity of these peptides, as demonstrated against both gram-positive and gram-negative bacteria, especially *E. coli* strains, underscores their potential as versatile agents capable of addressing a wide array of pathogenic challenges. Similarly, Mishra et al. [5] found that FK-16 had strong antimicrobial activity against a wide range of bacteria, including both gram-positive and gram-negative species. Our study extends these findings by emphasizing the peptide’s efficacy against biofilm-associated pathogens found in orthopedic infections, particularly *S. epidermidis*. Wang et al. [13] highlighted the effectiveness of FK-16 in disrupting bacterial membranes, which is a mechanism of action that likely contributes to its broad-spectrum antimicrobial properties. Our findings align with this mechanism, suggesting that membrane disruption is a key factor in the efficacy of FK-16 and GF-17. Additionally, for GF-17, studies such as that of Wang et al. [16] demonstrated its enhanced stability and reduced toxicity compared to longer peptides, making it a promising candidate for therapeutic applications. 

When comparing the tested AMPs with positive controls such as vancomycin, differences were observed. In the preliminary studies on biofilm removal presented in this work, FK-16 and GF-17 demonstrated higher efficacy in killing *S. epidermidis* in biofilms compared to vancomycin, which showed structural disruption in almost all tested biofilms but was less effective against *S. epidermidis* GOI1153754-03-14. Specifically, FK-16 and GF-17 treatments resulted in the presence of more dead bacteria in *S. epidermidis* biofilms than vancomycin, although this difference was not statistically significant (p>0.05). Conversely, vancomycin was more effective in killing *S. aureus* strains in biofilms compared to FK-16 (Sau89 *p* < 0.05 and ATCC 49,230 *p* < 0.01). These results indicate that while FK-16 and GF-17 have superior performance in certain biofilm-associated infections, vancomycin remains more effective against others, highlighting the potential for tailored therapeutic approaches based on the specific bacterial strain involved.

Despite the promising results demonstrated by FK-16 and GF-17 peptides in inhibiting and killing orthopedic infection-relevant bacteria, this study delineates some limitations. One significant concern is the cytotoxicity observed at higher peptide concentrations. This phenomenon indicates a potential risk of adverse effects in clinical settings where high doses might be required to achieve therapeutic levels, especially in scenarios involving systemic administration. However, research by Zhang et al. [21] investigated the hemolytic and cytotoxic activities of GF-17, finding that while the peptide did exhibit cytotoxic effects at higher doses, its therapeutic window was acceptable for further development. Mishra et al. [30] also explored modifications to FK-16 to reduce its cytotoxicity while maintaining or enhancing its antimicrobial potency. Overall, this necessitates an optimization of the peptide structure and dosage to enhance their therapeutic index to make them safe for human use. 

Further complicating their potential integration into treatment protocols is the absence of synergistic effects with established antibiotics. Our findings suggest that these peptides, while effective on their own, do not enhance the activity of vancomycin against bacterial strains. This could be a limitation since synergism often allows for lower doses of each drug to be used, potentially reducing the risk of side effects. Thus, assessing synergistic effects with a broader range of antibiotics and other antimicrobial agents could provide more comprehensive data on potential combination therapies. However, the antimicrobial potential of these peptides allows for their use as standalone treatments, probably requiring additional validation and regulatory approval processes essential for the successful translation of antimicrobial peptides from bench to bedside. Thus, more combinations need to be assessed with different AMPs and antibiotics to explore the possibility of other synergistic effects. As standalone treatments, integrating these AMPs into biomaterials used in orthopedic surgeries, such as bone cements and prosthetic coatings, could offer innovative approaches to prevent infections at surgical sites, as supported elsewhere [5]. Such applications could be particularly valuable in settings where biofilm formation significantly complicates the treatment and outcomes of orthopedic procedures. Indeed, studies have explored LL-37 and its shorter analogs in various formulations, including FK-16, investigated for their potential use in topical treatments for skin infections and wound healing due to their strong antimicrobial activity and ability to disrupt biofilms [31]. Thus, advancing this line of research regarding coating agents could significantly impact the management of orthopedic infections, addressing both antimicrobial resistance and the need for new therapeutic strategies.

This study is particularly innovative as it extends the application of LL-37-derived peptides into the field of orthopedics, a domain where biofilm-related infections pose significant clinical challenges. Compared to previous studies [18,19,20,21], our research emphasizes the practical implications of using these peptides in a new and critical area of medicine. Firstly, we focus on the development and testing of LL-37-derived peptides FK-16 and GF-17, demonstrating their potential efficacy against orthopedic infections, particularly those involving *Staphylococcus epidermidis*. Unlike previous studies, our research highlights the significant in vitro antimicrobial activity of these peptides against both reference and clinically relevant strains associated with orthopedic implants. Furthermore, we considered the challenge of biofilm-associated infections, a critical issue in orthopedic surgery, by demonstrating the AMP’s ability to disrupt pre-formed in vitro biofilms and combat multidrug-resistant bacteria. This comprehensive approach underscores the potential of FK-16 and GF-17 as promising candidates for enhancing infection control in orthopedic procedures.

## 4. Materials and Methods

### 4.1. Experimental Design

This study started with the synthetic production and purification of the AMPs. Subsequently, a first screening was undertaken to assess the AMPs’ antimicrobial activity against ATCC standard bacterial strains by determining the MIC and MBC. This screening allowed us to exclude the less performant AMPs from successive tests. Selected AMPs were then assessed for their cytotoxicity and hemolysis. AMPs with strong cytotoxicity or hemolysis were also withdrawn from further analysis. Selected AMPs underwent other in vitro assays for further characterization: MIC and MBC on orthopedic infection clinical isolates, checkerboard assay for the assessment of synergy with antibiotics, selection of resistance assay to check whether bacteria were able to develop resistance against the AMPs and antibiofilm activity.

### 4.2. AMPs Synthesis and Purification

All of the generated peptides were chemically synthesized using the standard Fmoc-based solid-phase synthesis technique on Rink amide MBHA resin (Novabiochem). Coupling was performed using dicyclohexylcarbodiimide (DCC, Molekula Americas, Lewisville, TX, USA) and 1-hydroxybenzotriazole (HOBt, Anaspec, Fremont, CA, USA) in the presence of a 5-fold excess of Fmoc-amino acids. After cleavage and deprotection with a mixture of TFA/H_2_O/triisopropylsilane (90:5:5, *v*/*v*/*v*) for 2 h at room temperature and evaporation of solvents, the crude mixture was repeatedly extracted/triturated with diethyl ether. After solvent removal, the crude peptide was purified by HPLC using reverse-phase column (Vydac C18, length: 250 mm, internal diameter: 20 mm, pore size: 300 Å, particle size: 15 mm) using an appropriate water/acetonitrile gradient in the presence of 0.1% TFA (Fisher Scientific, Milan, Italy). The purity (>95%) of the synthetic peptides was assessed by HPLC using an analytical reverse-phase column (Vydac C18, length: 250 mm, internal diameter: 4.6 mm, pore size: 300 Å, particle size: 5 mm). The characterization of peptides was confirmed by analysis using high resolution mass spectroscopy (LTQ-Orbitrap XL, Thermo Fisher Scientific, Milan, Italy).

### 4.3. Bacterial Strains and Culture Conditions

A total of 18 bacterial strains relevant to orthopedic infections were used for microbiological tests, including 8 ATCC (American Type Culture Collection, ATCC, Manassas, VA, USA) and 10 clinical isolates (CI) from the Laboratory of Clinical Chemistry and Microbiology, IRCCS Ospedale Galeazzi–Sant’Ambrogio of Milan (Italy) strain collection. The ATCC strains were used for the first screening (MIC/MBC) of all the AMPs produced, while the clinical isolates were used for further MIC/MBC assays on selected AMPs. Among CI, *S. epidermidis* GOI1153754-03-14 has been already characterized and used in previous studies [32,33]. The complete list of bacteria and their provenience and resistance are shown in Table 5. All the strains were stocked at −80°C and working stocks were stored at −20 °C. At the occurrence, the bacteria were resuscitated in Brain Hearth Infusion (BHI, Millipore, Milan, Italy) broth at 37 °C for 18 h from the working stocks.

### 4.4. Minimum Inhibitory Concentration (MIC) and Minimum Bactericidal Concentration (MBC)

To determine the minimum inhibitory concentration (MIC) and the minimum bactericidal concentration (MBC), ISO20776-1 and Clinical and Laboratory Standards Institute guidelines were followed [34,35]. A commercial synthetic LL-37 (Aurogene, Rome, Italy) was used as the control in the MIC and MBC assays. A stock solution of each AMP was prepared at 600 µg/mL to achieve a final concentration of 300 µg/mL. Additionally, to test the potential antimicrobial effect of the Trifluoroacetic acid (TFA), a Na-TFA (Fisher Scientific, Milan, Italy) stock solution of 400 µg/mL was also prepared, achieving a final concentration of 200 µg/mL, which was comparable in excess to the maximum concentration of TFA present in the commercial LL-37 (17 molecules TFA/molecule LL-37). The MIC was determined according to the aforementioned standard guidelines. The MBC was determined by plating 2 µL of culture from each well of the MIC plates on a Tryptic Soy Agar (TSA, Millipore, Milan, Italy) plate and incubated for 24 h at 37 °C. The concentration at which there was no growth was recorded as MBC. Each experiment was carried out in triplicate. Representative MIC and MBC values for vancomycin and gentamicin were assessed to offer a benchmark comparison for the AMPs. Quality control was conducted with *Staphylococcus aureus* ATCC 29,213 and vancomycin according to EUCAST guidelines.

### 4.5. Citotoxicity on Eukaryotic Cells 

The biocompatibility of AMPs was studied to exclude any cytotoxic effect on eukaryotic cells. NIH-3T3 murine fibroblasts (ATCC® CRL-1658, American Type Culture Collection—ATCC, Manassas, VA, USA) were used in this study. After cell thawing and expansion at 5 × 10^3^ cells/cm^2^, 5 × 10^4^ cells/cm^2^ were seeded in 96-well plates and cultured in complete medium (CM) consisting of Dulbecco’s Modified Eagle’s Medium (DMEM, ATCC® 30-2002, American Type Culture Collection—ATCC, Manassas, VA, USA), 10% calf bovine serum (ATCC® 30-2030™, American Type Culture Collection—ATCC, Manassas, VA, USA) and 100 U/ml penicillin–streptomycin (all from Gibco, Life Technologies, Monza, Italy). After 24 h of incubation at 37°C and 5% CO_2_, a solution of selected peptides in CM was added to the wells at final concentrations of 300, 150, 75, 37.5, 18.75, 9.38, 4.7 and 2.3 µg/mL. Cells cultured in fresh CM served as the negative control (NC), and fibroblasts cultured in CM supplemented with 0.1% Triton-X100 (Sigma-Aldrich, Merck, Milan, Italy) were used as the positive control (PC). After 24 h, cell viability was assessed using the MTT assay [36]. A microplate reader (Victor X3, Perkin Elmer, Milan, Italy) was used to read the absorbance at 570 nm. Two independent experiments were run in triplicate for each experimental condition. All the experimental procedures were carried out in the dark.

### 4.6. Hemolytic Activity Assessment

Fresh Human Blood Pool from 3 donors was purchased in K2EDTA tubes (CUST-BB-05052023-4A, CliniSciences s.r.l., Guidonia Montecelio, Italy) (2 mL), immediately mixed with 6 mL of Phosphate Buffered Saline (PBS, Gibco, Life Technologies, Monza, Italy) and centrifuged at 700× *g* for 8 min at room temperature (RT). The pellet containing erythrocytes was washed 3 times in PBS centrifuging at 700× *g* for 8 min at RT. Finally, 40 µL of pellet containing red blood cells (RBC) was diluted in 8 mL of PBS, obtaining a 0.5% *v*/*v* RBC suspension.

In a 96-well V plate, 75 µL of PBS was added to each well, and 75 µL of selected AMPs were serially diluted at 300, 150, 75, 37.5, 18.75, 9.38, 4.7 and 2.3 µg/mL, and finally 75 µL of RBC suspension were added to each well. The 96-well V plate was incubated at 37 °C for 1 h, then centrifuged at 1000× *g* for 10 min, and the supernatant (60 µL) was transferred to a 96-well flat bottom microplate for the absorption analysis on a microplate reader at 414 nm. The positive control (PC, 100% hemolysis) consisted of 0.1% Triton-X100 in PBS (pH 7.4), while PBS alone served as the negative control (NC, 0% hemolysis). Each hemolysis activity assessment test was carried out in two replicates and repeated three times.

NCs and PCs were averaged, then results were normalized to the average of PC and NC according to the following formula: % Hemolysis = (Abs treated − Abs NC/Abs PC − Abs NC) × 100. The hemolytic activity was also tested on TFA to exclude any toxicity caused by the buffer.

### 4.7. Checkerboard Assays to Assess the Presence of Synergy between Selected AMPs and Vancomycin 

A checkerboard assay was performed to determine the synergistic effect of the combination of AMPs and vancomycin (Pharmatex, Milan, Italy) in comparison to their own single activities by determining the fractional inhibitory concentration (FIC) index of all combinations tested as described elsewhere [37]. The FIC index was calculated as follows: FIC index = FIC #1 + FIC #2, where FIC #1 is the MIC of molecule #1 in the combination/MIC of molecule #1 alone, and FIC #2 is the MIC of molecule #2 in the combination/MIC of molecule #2 alone. The combination was considered synergistic when the FIC index was ≤0.5, additive when the FIC index was between 0.5 and 1, indifferent when the FIC index was between 1 and 2 and antagonistic when the FIC index was ≥2. Each combination was tested against four bacterial strains. Each experiment was carried out in triplicate.

### 4.8. Selection for Resistance

The emergence of bacterial resistance to the selected AMPs was investigated using a modified Kirby–Bauer method [38]. Briefly, a sterile 10-mm filter paper disk soaked in a solution of AMP 3 mg/mL was placed in the middle of a tryptic soy agar (TSA) plate seeded with approximately 1.5 × 10^8^ CFU/mL and incubated for 24 h at 37 °C. The following day, the zone of inhibition (ZOI) was measured, and bacteria grown on the edge of the ZOI were resuspended and seeded on another plate with a new AMP disc. The process was repeated for a period of 7 days (T7). At T0 and T7, the broth microdilution method was used to determine the MIC. If resistance was detected, the resistant strains were seeded in fresh TSA plates without AMP for further 7 consecutive days (T14) to assess if the acquired resistance to AMPs was stable or transient. The MIC was also assessed at T14 in this case. Acquisition of resistance was defined as a >4-fold increase in MIC. The eventually selected resistant strains were stored in BHI with 10% glycerol at −80 °C for further analyses. Each experiment was carried out in duplicate.

### 4.9. Biofilm Removal Activity

Rough titanium disks (Adler Ortho, Cormano, Milan, Italy) were autoclaved at 121°C for 15 min and placed into sterile 6-well plates. *S. epidermidis* ATCC 35984, *S. epidermidis* GOI1153754-03-14, *S. aureus* ATCC 49,230 and *S. aureus* Sau89 were grown overnight in BHI broth at 37 °C. The culture was diluted up to 1.5 × 10^6^ in BHI broth and 6 mL of each culture was inoculated in the disk containing wells. Two disks were inoculated with sterile BHI as sterility control. All the plates were incubated at 37 °C for 72 h. After incubation, the disks were gently washed with sterile saline to remove unattached cells. Each disk was treated with either vancomycin at 0.5% (Pharmatex, Milan, Italy), GF-17 (18.75 µg/mL) or FK-16 (75 µg/mL) for 18 h. Two disks for each bacterial inoculum were untreated as control. Subsequently, disks were gently washed with sterile saline solution and stained with Filmtracer^TM^ LIVE/DEAD^TM^ Biofilm Viability Kit (Thermo Fisher Diagnostics, Monza, Italy), according to the manufacturer’s instructions, washed again with sterile saline and observed with an upright TCS SP8 (Leica Microsystems CMS GmbH, Mannheim, Germany) using a 20X dry objective (HC PL FLUOTAR 20X/0.50 DRY). The images were acquired with Las X (Leica Microsystems CMS GmbH, Mannheim, Germany) and analyzed with Fiji software (Fiji, ImageJ version 1.53t, Wayne Rasband National Institutes of Health). For each channel, the biofilm area was measured after setting the threshold at 14 for the red channel and 20 for the green channel. The biofilm data were expressed as the percentage of red signal (dead cells) relative to the green channel (live cells). Finally, the data were normalized against the control, which was set to 1.

### 4.10. Data Analysis

Raw data were collected on Excel files. Graph Pad Prism 8.0 was used for data visualization and statistical analysis. One-way ANOVA with Tuckey’s multiple comparison test was used to assess differences (*p* < 0.05) between the AMPs’ antimicrobial activity. MIC and MBC values >300 µg/mL were arbitrarily considered as =300 µg/mL in the statistical analysis. Kruskal–Wallis with Dunn’s multiple comparison tests were used to assess statistical significance between the antibiofilm efficacy of FK-16, GF-17 and vancomycin.

## 5. Conclusions

This study confirms the potent antimicrobial activity of FK-16 and GF-17 and puts these findings within the broader context of current antimicrobial research. It highlights the crucial role of AMPs in addressing some of the most pressing issues in infection control, particularly those associated with orthopedic procedures where implant-related infections can lead to serious complications. The ability of these peptides to disrupt biofilms and combat antibiotic-resistant strains offers a promising avenue for developing more effective treatment protocols, potentially revolutionizing the management of infections in orthopedic and other medical fields. Furthermore, in vivo efficacy studies are planned using animal models of orthopedic infection induced with clinically isolated biofilm-forming bacteria. These future studies will aim to validate the therapeutic potential of FK-16 and GF-17 in a real scenario, paving the way for their application in clinical settings.

## Figures and Tables

**Table 1:** LL-37 and derived peptides with synthesized relative sequences and molecular weights.

Peptide	Sequence	Molecular Weight
**LL-37**	LLGDFFRKSKEKIGKEFKRIVQRIKDFLRNLVPRTES	4493.3
KR-12	KRIVQRIKDFLR	1571.9
FK-13	FKRIVQRIKDFLR	1719.1
FK-16	FKRIVQRIKDFLRNLV	2045.5
GF-17 ^a^	GFKRIVQRIKDFLRNLV	2102.5
17BIPHE2 ^a^	GBKR{D-LEU}VQR{D-LEU}KDB{D-LEU}RNLV	2101.5
SK-24	SKEKIGKEFKRIVQRIKDFLRNLV	2944.6

^a^ amidated at C terminus.

**Table 2:** MIC and MBC results of the commercial LL-37 (cLL-37), the six synthetic AMPs and Na-TFA against ATCC strains.

		Minimum Inhibitory Concentration (MIC)									
		AMP (µg/mL)									
Bacteria	Strain	cLL-37	Van	Gen	KR-12	FK-13	FK-16	GF-17	17BIPHE2	SK-24	Na-TFA
*S. epidermidis*	14990	75.00			>300.00	150.00	4.69	4.69	18.75	18.75	>200.00
*S. epidermidis*	35984	9.38			>300.00	150.00	9.38	2.34	37.50	37.50	>200.00
*S. aureus*	25923	37.50	0.6S	0.6S	>300.00	300.00	18.75	2.34	300.00	150.00	>200.00
*S. aureus*	43300	75.00			>300.00	300.00	9.38	4.69	150.00	150.00	>200.00
*S. aureus*	49230	75.00			>300.00	>300.00	18.75	4.69	300.00	75.00	>200.00
*P. aeruginosa*	27853	37.50	>40R	0.6S	>300.00	300.00	150.00	18.75	75.00	150.00	>200.00
*E. coli*	25922	37.50	>40R	0.6S	>300.00	75.00	18.75	9.38	37.50	75.00	>200.00
		**Minimum Bactericidal Concentration (MBC)**									
		**AMP (µg/mL)**									
**Bacteria**	**Strain**	**cLL-37**	**Van**	**Gen**	**KR-12**	**FK-13**	**FK-16**	**GF-17**	**17BIPHE2**	**SK-24**	**Na-TFA**
*S. epidermidis*	14990	75.00			>300.00	150.00	9.38	75.00	300.00	150.00	>200.00
*S. epidermidis*	35984	>300.00			>300.00	150.00	18.75	4.69	75.00	300.00	>200.00
*S. aureus*	25923	>300.00	0.6	2.5	>300.00	>300.00	18.75	37.50	>300.00	>300.00	>200.00
*S. aureus*	43300	>300.00			>300.00	>300.00	75.00	150.00	300.00	>300.00	>200.00
*S. aureus*	49230	>300.00			>300.00	>300.00	37.50	75.00	>300.00	>300.00	>200.00
*P. aeruginosa*	27853	75.00	>40	2.5	>300.00	300.00	300.00	75.00	150.00	150.00	>200.00
*E. coli*	25922	75.00	>40	2.5	>300.00	150.00	75.00	300.00	37.50	75.00	>200.00

**Table 3:** MIC and MBC results of cLL-37, FK-16 and GF-17 against 10 clinical isolates.

	Minimum Inhibitory Concentration (MIC)					
Microorganism Tested				AMP (µg/mL)		
Bacteria	Strain	Van	Gen	cLL-37	FK-16	GF-17
*S. epidermidis*	GOI1153754-03-14	1.25S	>20R	75.00	9.38	2.34
*S. aureus*	Sau39			150.00	4.69	2.34
*S. aureus*	Sau89			>300.00	18.75	4.69
*S. aureus*	Sau221			>300.00	18.75	9.38
*P. aeruginosa*	Pae2			75.00	150.00	150.00
*P. aeruginosa*	Pae61			37.50	150.00	37.50
*P. aeruginosa*	Pae82			37.50	150.00	75.00
*E. coli*	Eco53			37.50	18.75	18.75
*E. coli*	Eco61			37.50	9.38	9.38
*E. coli*	Eco75			37.50	18.75	18.75
	**Minimum Bactericidal Concentration (MBC)**					
**Microorganism Tested**				**AMP (µg/mL)**		
**Bacteria**	**Strain**	**Van**	**Gen**	**cLL-37**	**FK-16**	**GF-17**
*S. epidermidis*	GOI1153754-03-14	2.5	>20	>300.00	18.75	37.50
*S. aureus*	Sau39			>300.00	75.00	9.38
*S. aureus*	Sau89			>300.00	75.00	4.69
*S. aureus*	Sau221			>300.00	75.00	18.75
*P. aeruginosa*	Pae2			75.00	300.00	150.00
*P. aeruginosa*	Pae61			150.00	300.00	150.00
*P. aeruginosa*	Pae82			75.00	150.00	300.00
*E. coli*	Eco53			300.00	18.75	75.00
*E. coli*	Eco61			75.00	37.50	18.75
*E. coli*	Eco75			300.00	37.50	75.00

**Table 4:** Checkerboard assay results. The FIC values are reported for each combination tested. FIC > 0.5 indicates no synergy.

	FK-16 + GF-17	GF-17 + Van	FK-16 + Van
*S. epidermidis* GOI1153754-03-14	0.65	1.02	2
*S. aureus* 43300	0.55	2	2
*S. epidermidis* 35984	0.77	2	2
*S. aureus* 29213	0.99	2	2

**Table 5:** Bacterial strains used for the microbiological tests. CI: clinical isolate. Source: source description. Resistance or predicted phenotype: antibiogram or in-silico resistance prediction.

Bacteria	Strain	Source	Resistance or Predicted Phenotype
*S. epidermidis*	ATCC 14990	Nose	Ampicillin, fosfomycin, tetracycline *
*S. epidermidis*	ATCC 35984	Catheter sepsis	Amikacin, gentamicin, tobramycin, streptomycin, spectinomycin, kanamycin, ampicillin, erythromycin, azithromycin, fosfomycin, penicillin *
*S. epidermidis*	GOI1153754-03-14 (CI)	Orthopedic infection	Benzyl penicillin/oxacillin/gentamicin/cefazolin/rifampin/levofloxacin
*S. aureus*	ATCC 25923	Wound	Susceptible *
*S. aureus*	ATCC 43300	Human clinical isolate	Amikacin, gentamicin, tobramycin, spectinomycin, ampicillin, erythromycin, azithromycin, penicillin *
*S. aureus*	ATCC 49230	Chronic osteomyelitis	No genome available *
*S. aureus*	ATCC 29213	Wound	Ampicillin *
*S. aureus*	Sau39 (CI)	Wound swab	Benzylpenicillin/gentamicin/levofloxacin
*S. aureus*	Sau89 (CI)	Synovial fluid	Benzylpenicillin/erythromycin/teicoplanin/fusidic acid
*S. aureus*	Sau221 (CI)	Biopsy	Gentamicin
*P. aeruginosa*	ATCC 27853	Blood culture	Kanamycin, ampicillin, amoxicillin/clavulanic acid, cefoxitin, ceftriaxone, chloramphenicol, Fosfomycin *
*P. aeruginosa*	Pae2 (CI)	Orthopedic infection	N/D
*P. aeruginosa*	Pae61 (CI)	Fixation device	Amoxicillin+clavulanic acid/cefazolin/cefotaxime/ceftriaxone/ciprofloxacin/levofloxacin/tigecyclin/trimetoprim
*P. aeruginosa*	Pae82 (CI)	Osteoarticular tissue	Cefotaxime/tigecyclin/fosfomycin/trimetoprim
*E. coli*	ATCC 25922	Human clinical isolate	Susceptible *
*E. coli*	Eco53 (CI)	Osteoarticular tissue	ESBL/cefotaxime/ciprofloxacin/levofloxacin
*E. coli*	Eco61 (CI)	Synovial fluid	ESBL/amoxicillin+clavulanic acid/ceftazidine/gentamicin/cefotaxime/ciprofloxacin/levofloxacin/trimetoprim
*E. coli*	Eco75 (CI)	Synovial fluid	Susceptible

* for ATCC strains, when a complete genome was available, resistance phenotypes were predicted using StarAMR.

## Data Availability

The raw data supporting the conclusions of this article will be made available by the authors without undue reservation. Available at: https://zenodo.org/records/12771924, accessed on 17 July 2024.
